# Linoleic Acid Intake and Physical Function: Pilot Results from the Health ABC Energy Expenditure Sub-Study

**DOI:** 10.20900/agmr20220001

**Published:** 2022-02-16

**Authors:** Martha A. Belury, Brian C. Clark, Ryan McGrath, Peggy M. Cawthon

**Affiliations:** 1 Department of Human Sciences, The Ohio State University, Columbus, OH 43210, USA; 2 Ohio Musculoskeletal and Neurological Institute, Ohio University, Athens, OH 45701, USA; 3 Department of Biomedical Sciences, Ohio University, Athens, OH 45701, USA; 4 Department of Geriatric Medicine, Ohio University, Athens, OH 45701, USA; 5 Department of Health, Nutrition, and Exercise Sciences, North Dakota State University, Fargo, ND 58108, USA; 6 Fargo VA Healthcare System, Fargo, ND 58102, USA; 7 California Pacific Medical Center Research Institute, San Francisco Coordinating Center, San Francisco, CA 94107, USA; 8 Department of Epidemiology and Biostatistics, University of California, San Francisco, CA 94158, USA

**Keywords:** activities of daily living, diet, epidemiology, food, polyunsaturated fat

## Abstract

**Background:**

Dietary fat quality is important for health and physical functioning in older adults. Linoleic acid is a dietary polyunsaturated fatty acid that is necessary for optimal inner-mitochondrial membrane function. However, limited evidence exists for examining the role of linoleic acid intake on indices of mobility and physical function. In this pilot study, we sought to examine the associations between linoleic acid intake and physical functioning in older adults.

**Methods:**

This secondary analysis of data from the Health, Aging, and Body Composition energy expenditure sub-study was conducted for our investigation. Ability to complete physical tasks such as climbing a flight of stairs, walking a quarter mile, and lifting 10 lbs. was self-reported. Daily linoleic acid intake was estimated from a food frequency questionnaire. Persons with daily linoleic acid intake below approximately 85% of Adequate Intake were considered as having low linoleic acid intake. Covariate-adjusted logistic models were used for the analyses.

**Results:**

The final analytical sample included 317 participants aged 74.4 ± 2.8 years who consumed 18.9 ± 11.4 g/day of linoleic acid, with 78 (24.6%) participants having low daily linoleic acid intake. Persons with low daily linoleic acid intake had 2.58 (95% confidence interval: 1.27–5.24) greater odds for a limitation in climbing stairs.

**Conclusions:**

Our pilot investigation found that low daily linoleic acid intake could be associated with physical function in older adults. Dietitians working with older patients may want to consider the importance of daily linoleic acid intake for health and certain physical function tasks.

## INTRODUCTION

Poor physical functioning is a hallmark precursor for functional disability in older adults. Practicing healthy lifestyle behaviors, such as maintaining diet quality, may help to preserve physical function during aging. For example, healthy dietary intake patterns and nutritional status are associated with reduced risk for functional disability [1]. Many dietary guidelines for optimizing human health include recommendations for dietary fat quality such as the need for polyunsaturated fatty acid and linoleic acid [2]. Linoleic acid is a required (essential) nutrient that abounds in edible oils, nuts and seeds [3].

Impaired mitochondrial function is associated with the decline of muscle function [4,5], and therefore, could be a therapeutic target for delaying the onset and progression of age-related mobility and physical function losses [6–8]. Mitochondrial capacity for synthesizing adenosine triphosphate (ATP) becomes less efficient as shown in ex-vivo and in-vivo assays [7,9,10], and chronic inflammation and insulin resistance additively worsen mitochondrial capacity [11–13]. Further, therapies that restore mitochondrial function to produce ATP can improve skeletal muscle health in older persons [9,10,14].

An intact inner-mitochondrial membrane made of the phospholipid, cardiolipin, is required for producing ATP. The optimal functional configuration of cardiolipin is enriched with four linoleic acid side chains. Linoleic Acid enriched cardiolipin provides the scaffold for the electron transport chain proteins to efficiently conduct ATP synthesis. Because humans cannot endogenously synthesize linoleic acid, diet is the sole source of linoleic acid to synthesize 4-linoleoyl-cardiolipin. During oxidative phosphorylation involving the electron transport chain, cardiolipin is remodeled by exchanging oxidized linoleic acid for new non-oxidized linoleic acid molecules. In rodent models, loss of 4-linoleoyl-cardiolipin and accumulation of linoleic acid-poor cardiolipin species in cardiac and skeletal muscles allow proton ‘leakage’, reduce ATP production [15–18] and impair cardiac or skeletal muscle function.

Several prospective cohort studies have found that higher linoleic acid, measured either as dietary intake or biomarker, was associated with a lower risk for cardiovascular-specific, cancer-specific, and early all-cause mortality [19]. In addition, linoleic acid levels were associated with lower ectopic lipids in muscle, reduced insulin resistance and risk for type 2 diabetes [20–23]. Randomized controlled trials that have evaluated the effect of dietary linoleic acid supplementation on cardiometabolic and physiologic outcomes revealed linoleic acid increased lean mass while reducing markers of inflammation and ectopic lipid content of the liver; however, intramuscular triglycerides were not measured [24–27]. Problematically, older adults have lower levels of serum linoleic acid (compared to younger peers) [28] and intakes might be decreasing as edible oils that were rich in linoleic acid are being replaced with oils containing minimal amounts [29].

Despite the theoretical framework supporting the importance of linoleic acid in muscle and mitochondrial health, the association of linoleic acid intake with physical mobility in older adults has not been well investigated. In this pilot study, we sought to examine the associations between linoleic acid intake and physical functioning in older adults.

## MATERIALS AND METHODS

### Participants

A secondary analysis of data from the Health, Aging, and Body Composition (Health ABC) energy expenditure sub-study was performed for this pilot investigation. In 1997–1998, 3075 participants aged 70–79 years from a random sample of white Medical beneficiaries and all age-eligible self-reported black community residents were invited by investigators from the University of Pittsburgh and University of Tennessee-Memphis to participate in the larger Health ABC study. To be included, at baseline participants must have reported no difficulty walking a quarter mile, climbing a flight of stairs, performing activities of daily living, no plans to leave their current geographic area of residence for the next 3 years, no life-threating illnesses [30,31].

The Health ABC completed an energy expenditure sub-study from 1998–2000. A randomly selected record of 500 participants distinguished by sex and race was created from eligible individuals. A replacement list of approximately 200 participants was also created. Persons of the same sex and race from the replacement list were contacted when a participant from the main list was ineligible [30,31]. After recruitment, there were 323 participants enrolled in the Health ABC energy expenditure sub-study [32]. More details about the Health ABC energy expenditure sub-study are available elsewhere [31,32]. Our analytic sample included 317 persons with information for physical function, linoleic acid, age, sex, race, body mass index, educational achievement, and cigarette smoking status. A data flow diagram is shown in Appendix 1. All participants provided written informed consent and protocols were approved by the University of Pittsburgh and University of Tennessee-Memphis Institutional Review Board. The analysis plan used for our study was approved by the Health ABC under reference #AP21-1550 [33].

### Measures

#### Functional limitations

Participants were asked about their ability to climb a flight of stairs, walk a quarter mile, and lift 10 pounds. Persons responding “a little difficulty”, “some difficulty”, “very difficult”, and “unable to do” for each task were considered as having a limitation in that individual task [34].

#### Linoleic acid

Daily linoleic acid intake (g) was determined from the Health ABC food frequency questionnaire [35]. Calculations for daily linoleic acid intake were estimated from the self-reported dietary intake information from the food frequency questionnaire. Participants with daily linoleic acid intake below 85% of Adequate Intake (<12.0 g for men and <9.6 g for women) were considered as having low linoleic acid intake [36].

#### Covariates

Age, sex (male, female), race (white, not-white), educational achievement (not a high school graduate, at least a high school graduate), and cigarette smoking status (current smoker, not a current smoker) were self-reported. Body mass and standing height were measured with standard procedures, and a body mass index of ≥30 kg per meters-squared was used for determining obesity status (present, absent).

### Statistical Analysis

All analyses were conducted with SAS Enterprise Guide 7.1 (SAS Institute; Cary, NC). Descriptive information was presented as mean ± standard deviation or frequency (percentage) for continuous and categorical variables, respectively. Means and 95% confidence intervals (CI) were compared for the descriptive characteristics of the participants by linoleic acid intake status. Mean and CI daily linoleic acid intake was similarly compared between persons with a presence or absence of having a limitation in climbing a flight of stairs, walking a quarter mile, and lifting 10 pounds. Individual crude and covariate-adjusted logistic regression models examined the associations between low daily linoleic acid intake and limitations in each physical function task (climbing a flight of stairs, walking a quarter mile, lifting 10 pounds). Age, sex, race, educational achievement, cigarette smoking status, and obesity status were included as covariates in the adjusted logit models. The covariates included in our fully-adjusted models were pre-specified by the investigators based on data availability and because they were thought to be influential for our associations.

We also conducted supplementary analyses. A correlation analysis examined the relationships between daily linoleic acid, protein, carbohydrate, fat, and caloric intake. The means and CI for daily linoleic acid intake and physical function were compared by obesity status and the median split of doubly labeled water measured daily energy expenditure. More details about how doubly labeled water measured daily energy expenditure was collected is available elsewhere [32]. Separate logistic regression models again evaluated the associations between low daily linoleic acid intake and limitations in individual physical function tasks. Each logit model included five different sets of covariates for examining how the additional covariates influenced the estimates: Model 1 adjusted age, sex, race, educational achievement, cigarette smoking status, obesity status, and absolute daily protein intake; Model 2 included the covariates from Model 1 and absolute daily carbohydrate intake; Model 3 included the covariates from Model 2 and absolute daily fat intake; Model 4 included the covariates from Model 3 and absolute daily energy intake; Model 5 included the covariates from Model 4 and daily energy expenditure. Finally, we created tertiles from daily linoleic acid intake, and individual fully-adjusted logistic regression models examined the associations between the daily linoleic acid intake tertiles (reference: middle tertile) and limitations in each physical function task. All supplementary analyses were presented as an appendix because they were not principal for our investigation. An alpha level of 0.05 was used for all analyses.

## RESULTS

Table 1 presents the descriptive characteristics of the participants. The sample were aged 74.4 ± 2.8 years and consumed 18.9 ± 11.4 g/day of linoleic acid. Table 2 shows the descriptive characteristics of the participants by linoleic acid intake status. Persons with low daily linoleic acid intake, defined as below ~85% Adequate Intake, had linoleic acid intake of 7.8 g (CI: 7.3–8.3) compared to individuals with adequate daily linoleic acid intake (22.5 g; CI: 21.1–23.9). Daily linoleic acid intake for persons not reporting a limitation in climbing a flight of stairs was 18.9 g (CI: 17.6–20.2), but was 18.5 g (CI: 13.7–23.3) for individuals with a limitation in climbing stairs. Participants not reporting a limitation in walking a quarter mile had a daily linoleic acid intake of 18.6 g (CI: 17.3–19.9), and daily linoleic acid intake was 19.9 (CI: 16.4–23.5) in persons with a limitation in walking a quarter mile. Daily linoleic acid intake was 19.0 g (CI: 17.6–20.4) and 17.7 g (CI: 14.7–20.7) for participants without and with a limitation in lifting 10 pounds, respectively.

Table 3 presents the results for the associations between linoleic acid intake and physical function. The crude logistic model revealed that persons with low daily linoleic acid intake had 2.45 (CI: 1.26–4.78) greater odds for a limitation in climbing stairs, and the odds ratio changed to 2.58 (CI: 1.27–5.24) after adjusting for relevant covariates. A non-significant association was observed for low daily linoleic acid intake and having a limitation in walking a quarter mile (crude odds ratio: 0.89 (CI: 0.46–1.73); adjusted odds ratio: 0.89 (CI: 0.44–1.77)) and lifting 10 lbs. (crude odds ratio: 0.56 (CI: 0.20–1.51); adjusted odds ratio: 0.55 (CI: 0.19–1.59)).

The results for the relationships between daily linoleic acid, protein, carbohydrate, fat, and energy intake are shown in Appendix 2. Daily linoleic acid intake was correlated with daily protein (*r* = 0.70; *p* < 0.001), carbohydrate (*r* = 0.63; *p* < 0.001), fat (*r* = 0.92; *p* < 0.001), and energy intake (*r* = 0.82; *p* < 0.001). Appendix 3 presents the means and CI for daily linoleic acid intake and physical function by obesity and energy expenditure status (median split = 8.81 megajoules). There were more persons with obesity that had a limitation in climbing a flight of stairs (25.0%; CI: 15.5%–34.4%) than without obesity (10.1%; CI: 6.2%–13.9%). Similarly, there were more participants with a limitation in lifting 10 lbs below the daily energy expenditure median split (14.5%; CI: 8.9%–20.2%) than those ≥ the median split (5.2%; CI: 1.7%–8.8%). Appendix 4 shows the results for the associations between low daily linoleic acid intake and physical limitations with the additional energy intake and expenditure covariates included. Low daily linoleic acid intake remained associated with limitations in climbing a flight of stairs even after individually including the additional energy intake and expenditure covariates to the models. The findings for the associations between the daily linoleic acid intake tertiles (low tertile ≤ 12.4 g, middle tertile = 12.5–20.5 g, high tertile ≥ 20.6 g) and physical limitations are presented in Appendix 5. Relative to participants with daily linoleic acid intake 12.5–20.5 g, persons with daily linoleic acid intake ≤12.4 g had 3.56 (CI: 1.39–9.13) greater odds for a limitation in climbing a flight of stairs, but those with daily linoleic acid intake ≥20.6 g were not significantly at greater odds for a stair climbing limitation (odds ratio: 2.35; CI: 0.89, 6.22).

## DISCUSSION

Our findings revealed that low linoleic acid intake could be related to physical functioning in older adults. Specifically, we found that low daily linoleic acid intake was associated with greater odds for a limitation in climbing stairs. Falls during climbing stairs are a common source of injury and death in older adults [37]. Of the physical function tasks evaluated in our pilot study, climbing stairs could be the most bioenergetically demanding. Given the physical challenges older adults may experience when climbing stairs, this may help to explain why our pilot results found that low daily linoleic acid intake was significantly associated with limitations in climbing stairs, but not the other functional tasks we examined.

Impaired mitochondrial functioning is associated with declines in physical function [38]. Tetralinoleoyl-cardiolipin is a phospholipid of the inner mitochondrial membrane that supports optimal mitochondrial respiration for making ATP. As the scaffold for the electron transport chain, tetralinoleoyl-cardiolipin undergoes remodeling whereby oxidized linoleic acid molecules are for new non-oxidized linoleic acid molecules. In pre-clinical models of muscle atrophy, dietary linoleic acid is required to maintain a steady state supply of linoleic acid for replenishing tetralinoleoyl-cardiolipin: when rats or mice were fed diets with low levels of linoleic acid, cardiac and skeletal muscle strength and function were impaired [15,39,40]. Cumulatively, these studies prompted the question of whether low dietary linoleic acid intake is associated with deterioration of physical function via a mechanism involving linoleic acid targeting tetralinoleoyl-cardiolipin, a hypothesis recently raised by others [41].

Older Americans are generally at an elevated risk for low linoleic acid intake, and low blood and tissue levels of linoleic acid [28,29,42]. Even with this evidence, a common misperception may exist such that the intake of linoleic acid (a main omega-6 fatty acid in diets) is too high. In fact, in this cohort, about a quarter of older adults were consuming linoleic acid below the Adequate Intake. In addition to linoleic acid intake being at risk for insufficient intake in older populations, the intake of omega-3 fatty acids (e.g., alpha-linolenic acid and long chain omega-3 fatty acids) is also below Adequate Intake or optimal levels, where American adults have a narrow range of dietary intake of omega-3 fatty acids. Therefore, we refrained from calculating an omega-6/omega-3 ratio in this study because it would be driven primarily by the low intake of omega-3 intake and may not add new information beyond other reported findings [43].

Some limitations should be noted. Although we were specifically interested in the role of linoleic acid for this pilot work, the authors acknowledge that linoleic acid is a single fatty acid, and that other micro and macronutrients, such as protein, not included in our principal analyses also contribute to physical functioning [44]. Linoleic acid intake was correlated with other energy intake variables, thereby making it challenging to discern the role of dietary linoleic acid intake and physical function. Daily linoleic acid intake and the physical function measures were self-reported and thereby subject to relevant biases. Physical activity was not considered for our analyses because the physical activity questionnaire included items that intersected with our response variable (e.g., walking). The inclusion criteria for the Health ABC study suggests that participants at baseline were relatively healthy, homogeneous, and wellperforming. Despite these limitations, our pilot study presents unique information about the association of linoleic acid intake and physical functioning in a sample of older adults.

## CONCLUSIONS

This pilot investigation found that low daily linoleic acid intake could be associated with physical function in older adults. Dietitians working with older patients may want to consider the importance of daily linoleic acid intake for health and certain physical function tasks. We suggest that rigorous trials be conducted to investigate the potential for linoleic acid dietary intake and supplementation to enhance physical function in older adults.

## Figures and Tables

**Table 1.** Descriptive Characteristics of the Participants.

Variable	*n* = 317

Daily Linoleic Acid Intake (g)	18.9 ± 11.4
Low Linoleic Acid Intake^‡^ (*n* (%))	78 (24.6)
Male (*n* (%))	158 (49.8)
White Race (*n* (%))	165 (52.0)
Obese (*n* (%))	80 (25.2)
Age (years)	74.4 ± 2.8
Not a High School Graduate (*n* (%))	87 (27.4)
Current Smoker (*n* (%))	35 (11.0)
Limitation in Climbing a Flight of Stairs (*n* (%))	44 (13.8)
Limitation in Walking a Quarter Mile (*n* (%))	61 (19.2)
Limitation in Lifting 10 lbs. (*n* (%))	31 (9.7)

‡ Daily linoleic acid intake below ~85% of Adequate Intake (<12.0 g for men and <9.6 g for women).

**Table 2.** Descriptive Characteristics of the Participants by Linoleic Acid Intake Status.

Variable	Low Daily Linoleic Acid Intake^‡^ (*n* = 78)	Adequate Daily Linoleic Acid Intake (*n* = 239)

Daily Linoleic Acid Intake (g)	7.8 (7.3, 8.3)	22.5 (21.1, 23.9)*
Male (%)	56.4 (45.4, 67.4)	47.7 (41.3, 54.0)
White Race (%)	50.0 (38.9, 61.1)	52.7 (46.3, 59.0)
Obese (%)	33.3 (22.8, 43.7)	22.5 (17.2, 27.9)
Age (years)	74.1 (73.4, 74.7)	74.5 (74.1, 74.9)
Not a High School Graduate (%)	23.0 (13.7, 32.4)	28.8 (23.1, 34.6)
Current Smoker (%)	10.2 (3.5, 16.9)	11.3 (7.2, 15.3)
Limitation in Climbing a Flight of Stairs (%)	23.0 (13.7, 32.4)	10.8 (6.9, 14.8)
Limitation in Walking a Quarter Mile (%)	17.9 (9.4, 26.4)	19.6 (14.6, 24.7)
Limitation in Lifting 10 lbs. (%)	6.4 (0.1, 11.8)	10.8 (6.9, 14.8)

* Non-overlapping confidence intervals between groups.

‡ Daily linoleic acid intake below ~85% of Adequate Intake (<12.0 g for men and <9.6 g for women).

*Note:* Results are presented as mean (95% confidence interval).

**Table 3.** Results for the Associations between Low Daily Linoleic Acid Intake and Physical Limitations.

Variable	Low Daily Linoleic Acid Intake^‡^	

	Crude Odds Ratio (95% Confidence Interval)	Adjusted Odds Ratio^†^ (95% Confidence Interval)

Limitation in Climbing a Flight of Stairs	2.45 (1.26, 4.78)	2.58 (1.27, 5.24)
Limitation in Walking a Quarter Mile	0.89 (0.46, 1.73)	0.89 (0.44, 1.77)
Limitation in Lifting 10 lbs.	0.56 (0.20, 1.51)	0.55 (0.19, 1.59)

‡ Reference: Daily linoleic acid intake below ~85% of Adequate Intake (<12.0 g for men and <9.6 g for women).

† Adjusted for age, sex, race, educational achievement, cigarette smoking status, and obesity status.

**Appendix 1.**
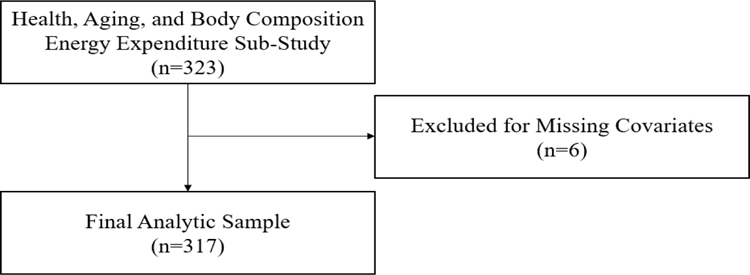
Data Flow Diagram.

## Data Availability

Data are available for download with approval at the Health ABC website (https://healthabc.nia.nih.gov/).

## APPENDICES

**Appendix 2.** Correlations Between Daily Linoleic Acid, Macronutrients, and Caloric Intake.

Variable	Linoleic Acid Intake (g)

Protein Intake (g)	*r* = 0.70*
Carbohydrate Intake (g)	*r* = 0.63*
Fat Intake (g)	*r* = 0.92*
Energy Intake (kcal)	*r* = 0.82*

* *p* < 0.001.

**Appendix 3.** Means and 95% Confidence Intervals for Daily Linoleic Acid Intake and Physical Limitations by Obesity and Energy Expenditure Status.

	**Obesity Status** ^†^	
	
	**Not Obese (*n* = 237)**	**Obese (*n* = 80)**
	
Daily Linoleic Acid Intake (g)	18.7 (17.3, 20.1)	19.4 (16.4, 22.4)
Low Daily Linoleic Acid Intake (%)	21.9 (16.6, 27.2)	32.5 (22.2, 42.7)
Limitation in Climbing a Flight of Stairs (%)	10.1 (6.2, 13.9)	25.0 (15.5, 34.4)
Limitation in Walking a Quarter Mile (%)	15.6 (10.9, 20.2)	30.0 (19.9, 40.0)
Limitation in Lifting 10 lbs. (%)	7.5 (4.2, 10.9)	16.2 (8.1, 24.3)
	
	**Median Daily Energy Expenditur** ^‡^	
	
	**< Median Split (*n* = 151)**	**≥ Median Split (*n* = 152)**
	
Daily Linoleic Acid Intake (g)	17.7 (16.0, 19.3)	20.1 (18.1, 22.1)
Low Daily Linoleic Acid Intake (%)	20.5 (14.0, 26.9)	26.9 (19.9, 34.0)
Limitation in Climbing a Flight of Stairs (%)	13.2 (7.8, 18.6)	14.4 (8.8, 20.7)
Limitation in Walking a Quarter Mile (%)	22.5 (15.8, 29.1)	17.7 (11.6, 23.8)
Limitation in Lifting 10 lbs. (%)	14.5 (8.9, 20.2)	5.2 (1.7, 8.8)

† Obese = body mass index ≥30 kg per meters-squared.

‡ Median Daily Energy Expenditure = 8.81 megajoules.

¥ There were *n* = 14 excluded due to missing data for energy expenditure.

**Appendix 4.** Results for the Associations between Low Daily Linoleic Acid Intake and Physical Limitations with Additional Energy Intake and Expenditure Covariates.

Variable	Odds Ratio (95% Confidence Interval)

*Model 1*	
Limitation in Climbing a Flight of Stairs	3.26 (1.47, 7.24)
Limitation in Walking a Quarter Mile	1.00 (0.47, 2.13)
Limitation in Lifting 10 lbs.	0.37 (0.12, 1.18)
*Model 2*	
Limitation in Climbing a Flight of Stairs	3.47 (1.53, 7.85)
Limitation in Walking a Quarter Mile	0.99 (0.46, 2.13)
Limitation in Lifting 10 lbs.	0.42 (0.13, 1.33)
*Model 3*	
Limitation in Climbing a Flight of Stairs	4.23 (1.70, 10.50)
Limitation in Walking a Quarter Mile	1.05 (0.46, 2.38)
Limitation in Lifting 10 lbs.	0.42 (0.12, 1.46)
*Model 4*	
Limitation in Climbing a Flight of Stairs	4.23 (1.70, 10.50)
Limitation in Walking a Quarter Mile	1.05 (0.46, 2.38)
Limitation in Lifting 10 lbs.	0.40 (0.11, 1.43)
*Model 5* ^†^	
Limitation in Climbing a Flight of Stairs	4.27 (1.68, 10.82)
Limitation in Walking a Quarter Mile	1.17 (0.51, 2.67)
Limitation in Lifting 10 lbs.	0.35 (0.09, 1.35)

† There were n=14 excluded in Model 5 due to missing data for energy expenditure.

*Note:* Model 1 adjusted for age, sex, race, educational achievement, cigarette smoking status, obesity status, and absolute daily protein intake; Model 2 adjusted for Model 1 + absolute daily carbohydrate intake; Model 3 adjusted for Model 2 + absolute daily fat intake; Model 4 adjusted for Model 3 + absolute daily energy intake; Model 5 adjusted for Model 4 + daily energy expenditure.

**Appendix 5.** Results for the Associations between the Daily Linoleic Acid Intake Tertiles and Physical Limitations.

Variable	Middle Daily Linoleic Acid Intake Tertile^‡^	

	Low Daily Linoleic Acid Intake Tertile Odds Ratio (95% Confidence Interval)^†^	High Daily Linoleic Acid Intake Tertile Odds Ratio (95% Confidence Interval) ^†^

Limitation in Climbing a Flight of Stairs	3.56 (1.39, 9.13)	2.35 (0.89, 6.22)
Limitation in Walking a Quarter Mile	0.89 (0.43, 1.82)	0.83 (0.40, 1.71)
Limitation in Lifting 10 lbs.	0.61 (0.23, 1.61)	0.66 (0.25, 1.74)

‡ Reference: Daily linoleic acid intake between 12.5–20.5 g.

† Adjusted for age, sex, race, educational achievement, cigarette smoking status, and obesity status.

*Note*: Low daily linoleic acid intake tertile is ≤12.4 g and high daily linoleic acid intake tertile is ≥20.6 g.

## ABBREVIATIONS

ABC: aging, and body composition
ATP: adenosine triphosphate
CI: confidence interval
